# Lithium and nephrotoxicity: a literature review of approaches to clinical management and risk stratification

**DOI:** 10.1186/s12882-018-1101-4

**Published:** 2018-11-03

**Authors:** J. Davis, M. Desmond, M. Berk

**Affiliations:** 1 Department of Renal Medicine, University Hospital Geelong, Rotary House, 325 Ryrie St, Geelong, VIC Australia; 2 IMPACT Strategic Research Centre, School of Medicine, Barwon Health, Deakin University, 75 Pigdons Road, Geelong, Australia; 3 Orygen, The National Centre of Excellence in Youth Mental Health, the Department of Psychiatry, and the Florey Institute for Neuroscience and Mental Health, University of Melbourne, Parkville, VIC Australia

**Keywords:** Lithium, Chronic kidney disease, Nephrotoxicity, Nephrogenic diabetes insipidus

## Abstract

**Background:**

Despite lithium being the most efficacious treatment for bipolar disorder, its use has been decreasing at least in part due to concerns about its potential to cause significant nephrotoxicity. Whilst the ability of lithium to cause nephrogenic diabetes insipidus is well established, its ability to cause chronic kidney disease is a much more vexing issue, with various studies suggesting both positive and negative causality. Despite these differences, the weight of evidence suggests that lithium has the potential to cause end stage kidney disease, albeit over a prolonged period.

**Methods:**

A search strategy for this review was developed to identify appropriate studies, sourced from the electronic databases EMBASE, PubMed (NLM) and MEDLINE. Search terms included lithium with the AND operator to combine with nephrotoxicity or nephropathy or chronic kidney disease or nephrogenic diabetes insipidus or renal and pathophysiology.

**Results:**

The risks for the development of lithium induced nephropathy are less well defined but appear to include the length of duration of therapy as well as increasing age, as well as episodes of over dosage/elevated lithium levels. Whilst guidelines exist for the routine monitoring of lithium levels and renal function, it remains unclear when nephrological evaluation should occur, as well as when cessation of lithium therapy is appropriate balancing the significant attendant mental health risks as well as the potential for progression to occur despite cessation of therapy against the risks and morbidity of bipolar disorder itself.

**Conclusion:**

This paper will elucidate on the current evidence pertaining to the topic of the clinical management of lithium induced nephrotoxicity and provide a guide for clinicians who are faced with the long-term management of these patients.

## Background

Lithium remains the most efficacious therapy for a significant proportion of patients with type 1 bipolar disorder, protecting against both depression and mania as well as being the only therapy known to reduce the risk of suicide in this patient population [1–3]. Despite these therapeutic advantages the use of lithium in the treatment of bipolar disorder has been decreasing, thought in part to be due to concerns about potential nephrotoxicity [4]. Whilst it is clear that lithium can induce nephrogenic diabetes insipidus (NDI), with reported prevalence ranges from 20 to 87% in various studies [5], the question about its ability to cause chronic kidney disease (CKD) is less clear. The incidence of end stage kidney disease (ESKD) due to lithium appears to be very low. ANZDATA from the year 2000 suggests that between 0.2 to 0.7% of all new ESKD cases for that year were attributed to lithium induced nephropathy [6]. The development of ESKD may also be associated with a significant latent period, with some studies suggesting that a period of around 27 years may be required to see significant nephrotoxicity occur [7]. In addition, there continues to be evidence which suggests that lithium may not increase the risk of CKD. A recently published population based study that have found that after adjusting for age, sex, and baseline estimated glomerular filtration rate (eGFR) there was no significant difference in the rate of decline between subjects on lithium and controls, and perhaps the observed differences in prior papers has been a result of methodological flaws [8]. Despite these variable findings within the literature, the predominant view is one of lithium having the ability to cause a chronic tubulointerstitial nephritis which leads to progressive CKD over a period of many years and ESKD in about 1.5% of long term lithium users [9]. Delineating the risk factors for the development of CKD, as well as providing clinicians with a guide as to the optimal management of patients on lithium with progressive CKD is challenging. This conundrum is doubly difficult given the potential for a ‘point of no return’ in the progression of lithium induced nephropathy as well as the risk of relapse of bipolar disorder if therapy is stopped [10]. This review paper aims to explore these pertinent questions.

## Methods

A search strategy for this review was developed to identify appropriate studies, sourced from the electronic databases EMBASE, PubMed (NLM) and MEDLINE. All relevant articles published between 1977 and January 2018 were included for analysis. There was no language restriction on articles. Duplicate studies were removed. The reference lists of articles were examined for additional studies which met the inclusion criteria. Search terms included lithium with the AND operator to combine with nephrotoxicity or nephropathy or chronic kidney disease or nephrogenic diabetes insipidus or renal and pathophysiology. Studies which examined the efficacy and safety of lithium and the subsequent risks for CKD in those with bipolar affective disorder were included and could be incorporate both randomised and non-randomised trials. Animal based studies and duplicates were excluded. The data was independently extracted from the included studies by the primary reviewer and collated within a Microsoft Word document and included general study characteristics and intervention design. The data extracted was verified by the other two co-authors with all discrepancies resolved through discussion and consensus.

### Lithium induced nephropathy

The earliest reports of lithium having a deleterious effect on renal function first appeared in the 1970s with a case series of patients with a chronic tubulointerstitial nephropathy on renal biopsy attributed to lithium [11]. Lithium induced nephropathy appears to be a slowly progressive disease. The average latency period from the initiation of lithium until the presence of ESKD is at least 20 years [5], and more than 80% of patients with lithium as the main cause of their ESKD have been on lithium for greater than 20 years [12]. Other studies have shown that after a mean treatment duration of 6.5 years only 4% of patients will have an elevation in their serum creatinine, whereas after 19 years of therapy this proportion increases to 12% of patients [6]. The incidence of ESKD felt to be due to lithium is very low, estimated to between 0.2 to 0.7% as based on 2000 ANZDATA [13, 14], however this still represents an almost eightfold risk of ESKD when compared to the general population [7, 15]. The rate of decline of eGFR has been suggested to average around 0.92 ml/min/1.73m^2^ per year of lithium treatment and appears to be higher in women when compared to men [16]. It is also higher in the elderly, those with a longer duration of lithium exposure, and higher cumulative lithium dose [10, 17, 18]. Even in the absence of ESKD, there is an increased incidence of stage 3 CKD in those patients on long term lithium when compared to the general population, with estimates suggesting anywhere from 21 to 55% of longer-term lithium users have eGFRs which fall into this category [19].

In contrast to the above literature which suggests a direct correlation between the use of lithium the subsequent risks of CKD and/or ESKD, there remains a proportion of the literature which is less suggestive of the proposed link. A recent meta-analysis suggested that while eGFR may be impaired by lithium therapy, such impairment is not clinically significant in the majority of patients and may be confounded by multiple other variables given that people with bipolar disorder have more medical comorbidities including the metabolic syndrome, and more adverse lifestyle risk factors that together may be a moderator of the link between lithium and renal dysfunction [20]. This was echoed by another paper which suggested that whilst increases in serum creatinine do occur, they are not necessarily associated with clinically relevant abnormalities in renal function [21, 22]. Another study, initially commenced as a 2-year randomised controlled trial followed by a single blind extension to assess the potential neuroprotective effects and safety profile of chronic low dose lithium therapy found no decrement in eGFR after 4 years of therapy between 61 patients randomly assigned to receive lithium or placebo [23]. However, this study itself noted that other observational and much longer-term studies have been the ones which suggest that eGFR will decrease with long term lithium use and may additionally have been underpowered to detect such a correlation [23]. Other groups have found no correlation between the duration of therapy and decreases in eGFR when corrected for age and other vascular risk factors and suggest that the majority of observed differences may be due to these other detrimental impacts on renal function [24]. One Swedish study suggested that with the use of lower serum levels of lithium in modern practice compared to the early years of lithium use the risk of ESKD is negligible [25], which is mirrored by other nationwide population based studies [26]. Clos et al. found that after adjustment for co-morbidities, other medication use, and episodes of lithium toxicity in a longitudinal cohort study they found no increase in the risk of renal dysfunction for those who have a baseline eGFR higher than 60 ml/min/1.73m^2^ [8]. The main criticism of the majority of these trials is the short duration, given that lithium induced nephropathy appears to be a slowly progressive disease over many years, hence such shortened studies may miss the longer-term decrements in eGFR.

### Nephrogenic diabetes insipidus

Lithium is considered to be one of the most common causes of acquired NDI, with rates of overt NDI being approximately 12% seen in those treated on lithium for 15 years and rates of lesser impairment, such as polyuria or impaired renal concentrating ability being seen in 19% and 54% respectively [10]. The development of overt NDI is characterised by polydipsia, the production of excessive amounts of urine, > 3000 ml in 24 h, and a dilute urine osmolality < 300 mOsm/kg [27]. The defect in urinary concentrating ability may be seen as early as 2 to 4 months after commencement of therapy with lithium, but it typically becomes more evident with longer durations of therapy [5]. It is usually reversible with the cessation of lithium therapy, but may become irreversible with prolonged therapy [20]. The time to when this defect may become irreversible has not been well established. Amiloride, a potassium sparing diuretic that targets distal tubule epithelial sodium channels (ENaC) is the most established therapy for lithium induced NDI [6]. It is more likely to be effective when there is a mild to moderate concentrating deficit as opposed to overtly established NDI [5]. Hydrochlorothiazide, another diuretic targeting the distal tubule, may also have efficacy but has a more limited evidence base [7].

### Risks for lithium induced nephropahty

A longer duration of therapy appears to be the most consistently implicated risk in those studies which suggest that lithium can cause CKD [5, 28], followed be increasing age [17]. A lower initial eGFR value is predictive of further decreases in eGFR, and this decline may be greater in women [16]. Other factors include the total cumulative lithium dose, other concomitant risks for CKD such as hypertension and diabetes mellitus, the use of other nephrotoxic medications such as angiotensin converting enzyme inhibitors (ACE-I), angiotensin II receptor blockers (ARB), non-steroidal anti-inflammatory agents (NSAIDs) and thiazide diuretics, prior episodes of lithium toxicity, and NDI [10, 29]. The possibility of individually increased susceptibility for lithium induced nephropathy, either through genetic or other environmental factors, is possible but at this stage has little conclusive evidence, meaning a high-risk subgroup is unable to be delineated at the commencement of therapy at this time [30]. Some of the identified risks for the development of NDI appear to be similar to those which may cause CKD, with duration of treatment, serum lithium levels, and the frequency of acute toxicity from lithium all being associated with an increased risk for NDI [5].

Lithium induced NDI also appears to have its own unique risk factors including non-responsiveness of the mood disorder to lithium and perhaps differences in formulation with the use of slow release preparations and twice daily dosing seeming to infer increased risk [31]. Early in the course of NDI the cessation of lithium can lead to improvements in the renal concentration deficits over a period of around a year [10]. However importantly once NDI is established it may also persist despite the cessation of lithium therapy suggesting that the prolonged use of lithium can lead to irreversible changes in renal structure and function [13] (Table 1).

**Table 1:** Risk factors for lithium induced nephropathy and their proposed mechanisms

Risk Factor	Proposed Mechanism
Duration of lithium therapy	Prolonged lithium exposure leading to irreversible structural changes within the kidney parenchyma
Age	Age related decline in eGFR, polypharmacy, medical comorbidity
Lower initial eGFR	Reduced nephron mass, background tubulointerstitial damage
Female gender	Unclear mechanism
Cumulative lithium dose	Prolonged lithium exposure leading to irreversible structural changes within the kidney parenchyma
Other concomitant CKD risks (hypertension, diabetes mellitus)	Concomitant tubulointerstitial damage, nephrosclerosis
Concomitant use of nephrotoxic medications	Disruption of tubulo-glomerular feedback, volume contraction, drug-interactions
Prior episodes of lithium toxicity	Higher lithium concentrations, induction of acute kidney injury with subsequent chronic damage
NDI	Volume contraction leading to elevated lithium concentrations, may be surrogate marker for morphological changes occurring within the kidney tubules

Factors which may be predictive of a poor prognosis include the degree of interstitial fibrosis on renal biopsy as well as the presence of heavy proteinuria, the latter not being a typical finding of lithium induced nephropathy [6]. A once daily dose of lithium, independent of whether the preparation is sustained release or immediate release, may confer a lower risk of renal insufficiency compared to regimes with multiple dosing requirements per day [32–34]. It is unclear whether the modern practice of lower serum levels of lithium can ameliorate the risk of lithium induced nephropathy, as there is some suggestion that individual susceptibility may remain at what is considered to be therapeutic levels [30]. Certainly one study has suggested that a lithium level of up to 0.8 mmol/L may be safe in elderly patients who do not have pre-existing CKD, in line with a report that suggested that lithium treated patients with and without CKD did not significantly differ in their lithium levels (0.58 vs 0.59 mmol/L) [35]. However, another paper has suggested that in elderly patients with pre-morbid CKD, even if lithium concentrations were maintained < 0.8 mEq/L there was an association between lithium use and a clinically important decline in eGFR [31]. Another study found that < 50% of patients on long term lithium therapy with a reduced creatinine clearance had a serum lithium concentration > 1 mmol/L at one time or more, suggesting that lithium induced nephropathy may occur in the absence of episodes of lithium intoxication [13]. Other evidence suggests that even one serum level of lithium > 1.0 mmol/L can cause a significant effect on eGFR up to 3 months after exposure, and lithium induced episodes of acute kidney injury have been associated with longer term risk for the development of CKD [36].

### Monitoring, nephrology referral and cessation of lithium therapy

General guidelines for the monitoring of renal function for those maintained on long term lithium therapy suggest measurement of the serum creatinine and eGFR at the initiation of lithium therapy and subsequently at three to six month intervals thereafter [3, 7], although it should be noted there is substantial heterogeneity between various guidelines [37]. Patients who are noted to have a rise in creatinine on three or more occasions, even if their eGFR is > 60 ml/min/1.73m^2^ require further evaluation, including a urinalysis for haematuria, proteinuria, a review of their medical history with particular attention paid to cardiovascular, urological and medication history, and blood pressure control and management. Overt proteinuria should be further quantified with a urine protein to creatinine ratio [38]. All patients as part of general care for both their bipolar disorder and potential renal disease, need baseline monitoring of their weight and height in order to calculate a body mass index, attention to lipid profile including both low and high density lipoproteins and triglycerides, fasting glucose, other baseline blood tests including complete blood count and measurement of liver function, calcium and thyroid function [10, 39]. Lithium levels are recommended to be monitored every 3 to 6 months or as otherwise clinically indicated by major international guidelines [10, 16, 39]. The recommended serum lithium levels for the maintenance of bipolar disorder are between 0.5 to 0.8 mmol/L, with higher levels of 0.8–1.2 mmol/L generally only recommended during the acute manic phase of the illness [28]. Symptoms suggestive of the development of nephrogenic diabetes insipidus, such as polyuria or polydipsia, require further evaluation with a paired urine and serum sodium and osmolality and measurement of a 24-h urine collection for both volume to allow quantification of the amount of urine generated as well as creatinine to assess completeness [10, 40].

There are several medications which can interfere both with serum lithium levels and renal function whose use needs to be closely monitored. Amongst these include the use of NSAIDs, ACE-Is, and thiazide diuretics [4, 10]. Loop and potassium-sparing diuretics are generally considered to cause less disruption to serum lithium levels [10]. Lithium may also promote its own retention given its ability to induce polyuria and subsequent volume depletion, highlighting the importance of ensuring adequate hydration and enquiring about the symptoms of decreased renal concentrating ability in lithium treated patients [28]. Moreover, given that the proximal tubule handles lithium in a similar fashion to that of sodium, conditions which promote sodium retention such as the hypervolemic states of cirrhosis or congestive cardiac failure promote an increase in the fraction of lithium which is reabsorbed leading to elevated lithium levels [28].

**Fig. 1:**
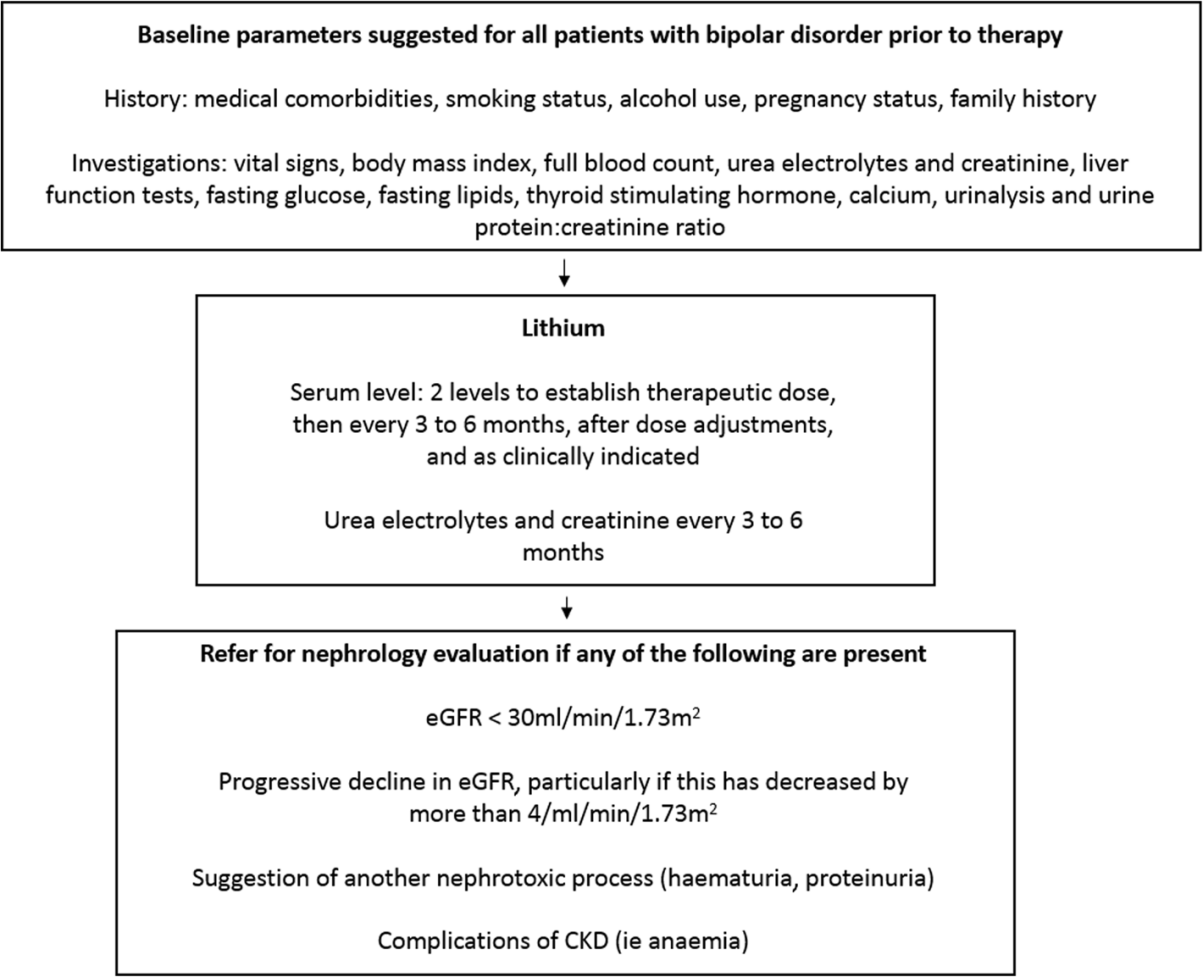
Suggested baseline parameters for all patients with bipolar disorder undergoing lithium therapy as well as ongoing monitoring and timing of referral. Adapted from the international society for bipolar disorder guidelines [10]

The timing of nephrology referral is a difficult question to answer. Whilst the 2012 Kidney Disease Improving Global Outcomes (KDIGO) guidelines would suggest referral when a patient’s eGFR is < 30 ml/min/1.73m^2^ [41] there is some evidence which suggests that this level of decreased eGFR in lithium treated patients may suggest irreversible renal damage, and individuals with lithium induced nephropathy are at risk for progression to ESKD despite discontinuation [42]. The exact point at which renal function may continue to deteriorate despite cessation of lithium is not known with certainty, but appears to lie around a creatinine clearance of up to 40 ml/min or a creatinine level of < 220 mmol/L [6, 43, 44], substantially higher than the recommended threshold for referral for nephrology evaluation (Fig. 1).

No systemic evidence exists to answer the question on when to stop lithium. There is no evidence that the performance of a renal biopsy changes clinical outcomes in lithium induced nephropathy, and like many other forms of slowly progressive CKD the performance of a renal biopsy rarely leads to a modification in therapy or the finding of a treatable disease [45]. Whilst there has been growing interest in the use of non-invasive imaging, particularly magnetic resonance imaging (MRI), for the visualisation of lithium induced microcystic disease as support for the clinical diagnosis of lithium induced nephropathy the utility of imaging for the routine clinical management of patients treated with long term lithium remains to be delineated [46, 47]. In addition, the significant impact on the patients mental health also needs to be considered [48]. For example, one would be less tempted to cease lithium in excellent lithium responders especially those who have not done well previously on other therapies, or whose illnesses have been severe or accompanied by risk to self or others. One would be more tempted to cease lithium if tolerability is poorer, there has been a history of response to alternatives or if the person does not have classical bipolar I disorder. Patients who are of a younger age, in whom the accelerated decline in eGFR with ongoing lithium exposure would mean they are proportionally more likely to reach ESKD during their remaining lifespan may also benefit from earlier consideration of lithium cessation. It should be noted that it is currently not clear for lithium based nephrotoxicity if early referral for nephrology services can halt the continuing decline in renal function [14], although evidence from early referral in other renal diseases suggest that the decline in eGFR can be slowed by early nephrology involvement [49]. General guidelines for referral for nephrology evaluation include a progressive decline in the eGFR, especially if this has decreased by more than 4 ml/min/1.73m^2^ per year, there is a suggestion of another nephrotoxic process occurring such as the finding of haematuria or significant proteinuria, there are potential complications of CKD such as anaemia, and eGFR < 30 ml/min/1.73m^2^ [38]. Given the above mentioned potential for lithium to have caused irreversible damage at higher levels of eGFR, there has been suggestions from some groups to refer for consideration of lithium discontinuation at much lesser degrees of renal dysfunction, with a creatinine level of 140 mmol/L proposed as one guide [7].

In regards to a decision making analysis of whether to cease lithium, concerns about relapse of otherwise stable bipolar disorder, as well as the potential for an increased risk of suicide with the use of alternative therapeutic options are significant and have to be balanced against the risk of potential future ESKD [43]. Stopping at an earlier stage of CKD may be appropriate if the mood disorder has been stable and another efficacious therapeutic option may exist for a particular patient and if there is clear evidence of a persistent decline in renal function over time with no other alternative cause found [48]. In addition, given the concerns that lithium induced nephropathy may continue to progress despite cessation of lithium and may even do so at a relatively innocuous creatinine clearance of < 40 ml/min or creatinine level of < 220 mmol/L [6], a time where patients would likely still be asymptomatic from their CKD, this rise in risk needs to be carefully considered in the decision-making algorithm. Alternatively, if the patient has had prior relapses and lithium is clearly the most efficacious medication then continuing with vigilant monitoring may be appropriate [48]. A process of shared informed decision making is essential, incorporating the person’s viewpoint on the available choices and their consequences. This is a decision best made gradually over time, in consultation with their treating psychiatrist, nephrologist, primary care physician and significant others if appropriate. If lithium is ceased, this should be done very gradually [50] and with close monitoring, together with understanding of early warning signs and engagement of caregivers if appropriate.

## Conclusions

Whilst lithium remains the most efficacious therapy for bipolar disorder there are some significant toxicities which may be associated with its long-term use. This difficulty is further compounded by the need of people suffering from bipolar disorder to take chronic therapy in order to prevent their risk of relapse. While the ability of lithium to cause CKD remains debated, the weight of evidence appears to favour its ability to do so. There remains no firm decision making algorithm to assist clinicians in the question of when to cease or if to cease lithium therapy in a patient with CKD, and the decision must be weighed against both the mental health of the individual and their risk of subsequent ESKD. Ultimately, a collaborative decision-making process between patient and healthcare provider may be the most useful discussion which may allow clinicians to guide their management.

## Abbreviations

ACE-I: Angiotensin converting enzyme inhibitor
ARB: Angiotensin II receptor blocker
CKD: Chronic kidney disease
eGFR: Estimated glomerular filtration rate
ESKD: End stage kidney disease
KDIGO: Kidney Disease Improving Global Outcomes
MRI: Magnetic resonance imaging
NDI: Nephrogenic diabetes insipidus
NSAID: Non-steroidal anti-inflammatory agent
